# Predictors of Satisfaction With Life and Health Status of Older People in Brunei: A Gender Comparative Study

**DOI:** 10.3389/ijph.2022.1605042

**Published:** 2022-08-25

**Authors:** Hanif Abdul Rahman, Armah Tengah, Yusnani Mohd Yusof, Ly Slesman, Chang-Yau Hoon, Khadizah H. Abdul-Mumin

**Affiliations:** ^1^ Centre for Advanced Research, Universiti Brunei Darussalam, Gadong, Brunei; ^2^ Pengiran Anak Puteri Rashidah Sa’adatul Bolkiah Institute of Health Sciences, Universiti Brunei Darussalam, Gadong, Brunei; ^3^ School of Nursing, University of Michigan, Ann Arbor, MI, United States; ^4^ School of Health Sciences, Politeknik Brunei, Bandar Seri Begawan, Brunei; ^5^ School of Nursing and Midwifery, La Trobe University, Bundoora, VIC, Australia

**Keywords:** older adults, predictors, gender, life satisfaciton, healthy ageing, Brunei

## Abstract

**Objective:** To investigate predictors of life satisfaction and healthy aging with focus on gender differences among older people in Brunei Darussalam.

**Methods:** A cross-sectional study on older people recruited by proportionate sampling. Multiple linear regression stratified by gender was applied.

**Results:** 45.6% of life satisfaction of older women were strongly associated with self-perceived health, social relationship, and education level. For older men, 26.3% of the variance of life satisfaction was predicted by physical functioning or disability, and social relationship. For older women, 38.9% of the variance of health status can be explained with satisfaction with life, and difficulty to do daily tasks. For older men, 33.1% of the variance of health status can be accounted by income, number of children, presence of chronic illness, and diabetes.

**Conclusion:** This paper discusses the unique gender differences of older people from a global perspective. Policymakers and stakeholders need to account for local and contextual differences before adopting international guideline. Particularly, on the maintenance or further promotion social interactions, active engaging elderly in health maintenance, and physical and mental functioning of the older population.

## Introduction

The world is rapidly aging as increasing longevity and decreasing fertility has become demographic trends [1, 2]. Research on aging has received considerable attention in medicine, public health and social sciences in recent decades. However, few had approached the importance of gender for successful aging [3]. It has become increasingly important to have gender-specific approaches to understand gender gaps among older adults. Prior evidences have suggested strong differences in disease distribution, clinical profiles, and socioeconomic status, among elderly men and women [4–7]. Aging population with higher chronic illnesses, disabilities, and dependency have significant economic and societal impact that will result in the rise of global burden of disease and disability [2, 8, 9]. There has been paramount efforts by government actors in collaboration with related stakeholders to increase period of “good health” and sustained sense of well-being to extend social productivity of older adults as much as possible [10].

Life satisfaction—a subjective feeling and attitude about one’s life at a particular point of time that could range from positive to negative—is one of the most important component of healthy aging [11]. Life satisfaction elicits a U-shaped pattern with age, meaning as a person grow older their overall satisfaction in life rises after a nadir at 50 years [12]. This corroborates with similar studies which demonstrated increasing life satisfaction with ageing [13–15] until it reaches a threshold at a certain point, which could differ by multitude of factors, and starts to decline [16, 17].

Successful aging is largely measured by subjective well-being, where life satisfaction is the major indicator [7]. There has been numerous studies investigating significant predictors of life satisfaction but gender-specific studies are scarce, which rendered limitations to enriching discussion and understanding of well-being and its determinants [7, 18–21]. Furthermore, prior evidence has established that healthy aging, is a strong contributor towards positive life satisfaction that affect elderly functioning and the primary focus of the current gerontological effort to achieve more positive outcome of aging [7].

Since the early 20th century, life expectancy has increased substantially and along with the concomitant decline in fertility rate, an ageing population is on the rise around the world. Brunei Darussalam (hereafter, Brunei) is no exception. A country is considered as an ageing population when 7 percent of the population are 65 years and above. According to the United Nation, Brunei’s older population will account for 7.7 per cent of the total population by 2025 [22]. This means in no time Brunei will have relatively higher proportion of its inhabitants being elderly compared to children [22]. By 2050, global life expectancy was estimated to increase by 4.5 years, an approximate six percent rise since 2015, and was predicted to increase continuously thereafter [22]. Still, questions remain as to whether these added years are experienced in good health and life satisfaction.

Therefore, this study was conducted with the purpose of investigating the predictors of life satisfaction and healthy aging with focus on gender differences.

## Methods

### Study Design

A cross-sectional study using interviewer-assisted questionnaire administration that was disseminated nationwide in all four districts of Brunei Darussalam.

### Participant Selection and Setting

The study was conducted in January 2019 to January 2020 in all four districts in Brunei where the targeted participants were those within the age range of 50–75 years; held Brunei citizenship or permanent residency; lived at home or resided in an institution; lived in a household; oriented to place and persons and able to communicate in English and Malay. Adults aged 50 years or below and those who were cognitively impaired were excluded. The survey was conducted at the home where respondents reside.

The cut-off age for older adults varies chronologically, socially and scientifically or functionally. There is no uniformity across the population of the aging process. Classification of age of the older adults are also not specific, mainly due to genetics differences, lifestyle, and overall health [23]. Although the World Health Organization and the United Nation defined older persons chronologically as those population aged 60 years onwards, uniquely in Brunei early retirement age is set at 55 years old indicating the start of older adults age in Brunei [24, 25]. Sociologically, the status of such as being grandparents, and the physical aging appearance may also be used to define older persons age [26]. Similarly, functionally, in medical and healthcare, when reporting age-related or chronic health conditions, older adults are often categorized as those aged 50 years of age onwards [27]. Due to this varying, confusing and complex definitions, for the purpose of this study, older persons are defined as those aged from 50 years of age.

The Ministry of Home Affairs via the District offices granted permission to access the current population census of the four districts in Brunei. All eligible participants (*n* = 63,900) were then sectioned out from the total study population (*n* = 76,000). Minimum sample size was calculated with precision of 5% (d = 0.05). A minimum sample of 385 was required to achieve precision of 0.5 at 95% confidence interval [28]. Stratified proportionate sampling was employed to ensure recruitment of participants were proportional to district and gender of study population.

### Data Collection Instruments

Data was collected by the appointed research assistants who were trained prior to data collections using a questionnaire adapted and developed from various validated and established tools including:

(1) Health and Retirement Study on Psychosocial and Lifestyle developed in the University of Michigan, United States (HRS) (Cronbach’s alpha ranged 0.77–0.89);
(2) English Longitudinal Survey on Aging (ELSA) jointly run by teams at University College London (UCL), the Institute for Fiscal Studies (IFS), NatCen Social Research and the University of Manchester in United Kingdom (Cronbach’s alpha ranged 0.89–0.90);
(3) Survey on Health, Aging and Retirement in Europe (SHARE) (Cronbach’s alpha range 0.69–0.88);
(4) Irish Longitudinal Study on Aging (TILDA) developed in Trinity College Dublin (Cronbach’s alpha 0.6–0.94);
(5) Korean Longitudinal Study of Aging (KLoSA) (Cronbach’s alpha 0.8–0.9);
(6) Japanese Study of Aging and Retirement (JSTAR) (Cronbach’s alpha 0.62–0.78).

A panel was formed to review the questions in the six questionnaires and decided to extract only relevant questions for the context of Brunei. The panel comprised of ten experts from different disciplines (Health, Economics, Business, Psychology, Social Studies, and Policy). All the relevant questions were compiled as one questionnaire that consists of:

(1) Demographics and family (8 items): Age of participants and spouse, marital status, living status, number of children, education level, and income level.
(2) Health status (20 items): overall health, satisfaction with life, ability to perform daily activities, and walking.
(3) Emotional conditions (16 items), social health (9 items), and physical health (12 items).

Each variable of interest (overall health and life satisfaction) was measured mainly using Likert-scale. For overall health, the item was scored from 1 = Very good to 4 = Poor. Satisfaction with current life was scored from 1 = Satisfied to 4 = Unsatisfied. The ability to perform daily activities items were measured dichotomously as Yes/No. The ability to walk during daily activities were scored from 1 = hardly walk to 6 = walk for more than 90 min. Emotional condition items were scored from 1 = Not at all to 4 = Always. Social health items were scored based on frequency (1 = Almost every day to every 2 months or more) and the number of persons involved (0 = None to 9 = 9 persons or more). Physical health was measured based on intensity of pain (0 = No pain to 4 = Severe pain).

The questionnaire was pre-tested on twenty elderly participants with the same inclusion criteria. Amendments to the questionnaire were made based on the feedback and comments given by the participants. In the actual data collection, two research assistants accompanied by a member of the research team collected the data. Participants were able to ask questions shall they require assistance. Full explanation of the study was offered and any questions regarding the study was answered prior to completing the questionnaire.

### Data Analysis

All analyses were stratified by elderly men and women. Descriptive statistics were used to describe the sample. Univariate analysis using Chi-square test for independence and Independent *t* test were applied, where indicated. Multiple linear regression estimation was performed for male and female sample. First, a stepwise automatic variable selection procedure based on the Akaike Information Criterion (AIC) was computed to select variables to be included in the regression model. The significant variables selected were then checked for interaction effects and their likely multicollinearity using Variance Inflation Factor (VIF). Furthermore, residual plots were then used to check for assumptions for overall linearity, linearity of each numerical independent variable, normality, and equal variance. “Standardized” residual plots were further used to check for outliers. Hosmer-Lemeshow goodness-of-fit test was used to check for the fit of final model. All statistical analyses were performed using RStudio v1.1.383. All statistical tests are two-sided and a *p-*value < 0.05 is considered statistically significant.

### Ethical Considerations

The research protocol of this study was reviewed by the Universiti Brunei Darussalam research ethics committee, which has approved that the present study conforms to ethical standards throughout the stages of the research process to ensure participants’ human rights were sufficiently safeguarded and protected (UBD/OAVCR/UREC/Apr18-04). Written informed consent was obtained from the participant prior to the study.

## Results

In total, 429 elderly persons participated in the study. Table 1 presents characteristics of the sample segregated by gender where 50.1% were male and 49.9% were female. A significant portion of the sample were recruited from Brunei-Muara district (77.4%, *p* = 0.029). Majority (about 94%) of the sample were of ethic Malay origin and of Islamic belief (96%). One in four (about 24%) of the participants were membership of an organisation, clubs or societies where they mostly had activity 2 to 3 times every week (male = 35.9% and female 64.1%).

**TABLE 1:** Descriptive statistics of participants’ characteristics by gender (National Study of Elderly Person, Brunei, 2019).

	Male (*n =* 215)		Female (*n =* 214)		*p-*value^a^
	*N*	(%)	*n*	(%)	
Marital status					
Married	195	(57.9)	142	(42.1)	**<0.001**
Others	20	(21.7)	72	(78.3)	
Living together with spouse?					
Each wife/husband living individual household	16	(66.7)	8	(33.3)	**<0.001**
All wives/husband living under one household	142	(78.0)	40	(22.0)	
Not specified or responded or refused	57	(25.7)	165	(74.3)	
Living together with household members?					
Yes	161	(48.6))	170	(51.4)	0.080
How many children live together with you?					
None	21	(35.6)	38	(64.4)	**0.018**
1 to 2 children	61	(45.2)	74	(54.8)	
3 to 4 children	73	(53.3)	64	(46.7)	
5 to 6 children	50	(62.5)	30	(37.5)	
>6 children	10	(55.6)	8	(44.4)	
Highest level of education attained					
No formal education/Primary school	11	(18.6)	48	(81.4)	**<0.001**
Lower secondary (Form 1–3)	58	(51.8)	54	(48.2)	
Upper Secondary (Form 4–5)	49	(43.0)	65	(57.0)	
Form 6	24	(68.6)	11	(31.4)	
Diploma	16	(61.6)	10	(38.5)	
Higher National Diploma	20	(74.1)	7	(25.9)	
Bachelor Degree	24	(66.7)	12	(33.3)	
Postgraduate Degree	13	(65.0)	7	(35.0)	
Estimated income (B$)					
250 and below	25	(22.9)	84	(77.1)	**<0.001**
251 to 500	15	(39.5)	23	(60.5)	
501 to 1,000	32	(60.4)	21	(39.6)	
1,001 to 1,500	53	(57.6)	39	(42.4)	
1,501 to 2000	31	(68.9)	14	(31.1)	
2,001 to 2,500	22	(59.5)	15	(40.5)	
2,501 to 3,000	17	(60.7)	11	(39.3)	
3,001 to 3,500	11	(78.6)	3	(21.4)	
3,501 and above	9	(69.2)	4	(30.8)	
Mean Age of participants in Years (SD)	60.1	(5.7)	60.9	(6.1)	0.144^b^
Mean Age of spouse in Years (SD)	57.8	(6.5)	63.1	(7.0)	**<0.001** ^**b**^

a = Chi-square test for independence; b = Independent test; n = count/frequency; SD, standard deviation.

Bold value represents significance at 0.05.

Univariate analysis showed that male participants were significantly higher in terms of smoking, married status, and living with spouse compared to female participants (*p* < 0.001). Female participants had significantly higher in terms of having 1 to 2 children. On the other hand, male participants had significantly higher in terms of having 3 or more children (*p* = 0.018). Male participants also had significantly higher educational attainment where it was observed that proportion of male was higher from Form 6 (pre-university) and above (*p* < 0.001). Male participants also had significantly higher estimated income where it was observed that proportion of male was higher from B$501 and above (*p* < 0.001).

Table 2 illustrates the health perception, life satisfaction, and ability to do daily activities. Univariate analysis showed that elderly men (60.6%) had significantly higher perception of “very good” health compared to elderly women (39.4%) (*p* < 0.001). This is similar for life satisfaction where 79.0% of elderly men and 65.4% of women reported “satisfied” with life. Nonetheless, both genders were equally concern regarding the importance of health (*p* = 0.740) where more than 80% had undergone health screening or medical checks.

**TABLE 2:** Health perception, Satisfaction with life and daily activities of participants by gender (National Study of Elderly Person, Brunei, 2019).

	Male (*n =* 215)		Female (*n =* 214)		*p-*value^a^
	n	(%)	n	(%)	
Overall current health					
Very good	83	(60.6)	54	(39.4)	**0.001**
Good	95	(50.5)	93	(49.5)	
Fair	30	(33.3)	60	(66.7)	
Poor or Don’t know	7	(50.0)	7	(50.0)	
Satisfaction with current life					
Satisfied	170	(54.8)	140	(45.2)	**0.008**
Fairly satisfied	37	(42.0)	51	(58.0)	
Somewhat satisfied to unsatisfied	8	(25.8)	23	(74.2)	
Performing everyday activities					
Difficulty using any vehicles by yourself	195	(57.7)	143	(42.3)	**<0.001**
Difficulty to shop for daily needs	199	(50.9)	192	(49.1)	0.387
Difficulty to shower on your own	198	(49.0)	206	(51.0)	0.102
Difficulty to go out and pay bills	195	(53.7)	168	(46.3)	**<0.001**
Difficulty to make deposits and withdrawal from bank	191	(54.7)	158	(45.3)	**<0.001**
Difficulty to collect your pension and so on	171	(55.5)	137	(44.5)	**0.001**
Difficulty to read any newspapers/books/magazines	180	(50.0)	180	(50.0)	1.000
Interested in articles or programs about health	178	(50.0)	178	(50.0)	1.000
Do you visit the homes of friends	151	(52.4)	137	(47.6)	0.205
Do you give advice to family or friends	191	(49.5)	195	(50.5)	0.530
Can you visit sick people?	198	(49.9)	199	(50.1)	0.865
Do you ever talk to young people?	190	(49.9)	191	(50.1)	0.892
Do you use telephone by yourself?	195	(50.9)	188	(49.1)	0.425
Do you take medicine by yourself?	192	(50.4)	179	(49.6)	0.878
Do you drive?	193	(60.5)	126	(39.5)	**<0.001**
Can you prepare your own meals?	190	(47.9)	207	(52.1)	**0.002**
Do you do gardening/fishing/or other hobbies?	150	(51.2)	143	(48.8)	0.581
Walking in the course of daily activities					
Hardly walk/cannot walk	12	(42.9)	16	(57.1)	0.106
Less than 30 min	28	(43.8)	36	(56.2)	
30–60 min	69	(47.9)	75	(52.1)	
61–90 min	26	(49.1)	27	(50.9)	
More than 90 min	80	(57.1)	60	(42.9)	

n = count/frequency.

a = Chi-square test for independence.

Bold value represents significance at 0.05.

In terms of performing daily activities, it was reported that current health problems did not impair physical functioning. Significantly higher male sample (61.8%) compared to female sample (38.2%) can still drive by themselves although they also had significantly higher report of difficulty when using vehicles (*p* < 0.001). They also reported significantly higher difficulty to go out and pay bills, make withdrawals or deposits, and collect pensions. On the other hand, female participants reported significantly higher difficulty in preparing meals for themselves.

Table 3 presents the mental and emotional conditions of participants as they currently perceived themselves compared to last week. The stratified analysis by gender revealed that emotional conditions among older adults were similar, in general. In conditions where there were significant differences, female participants exhibited higher emotional problems as compared to the past week, particularly in: “feeling frightened,” “feeling lonely,” “feel like crying,” “feeling sad,” “difficulty concentrating what I was doing,” and “something normally effortless became difficult to do.”

**TABLE 3:** Mental and Emotional conditions of participants last week by gender (National Study of Elderly Person, Brunei, 2019).

	Male (*n =* 215)		Female (*n =* 214)		*p-*value^a^
	n	(%)	n	(%)	
Emotional condition last week					
1. Felt depressed					
Not at all	180	(42.9)	161	(57.1)	0.083
Sometimes/Always	35	(39.8)	53	(60.2)	
2. Felt could not do a normal person could do					
Not at all	30	(60.0)	20	(40.0)	0.282
Sometimes	28	(41.8)	39	(58.2)	
Always	147	(50.3)	145	(49.7)	
Not applicable	10	(50.0)	10	(50.0)	
3. Could not concentrate what I was doing					
Not at all	147	(56.5)	113	(43.5)	**0.012**
Sometimes	29	(41.4)	41	(58.6)	
Always	31	(39.2)	48	(60.8)	
Not applicable	8	(40.0)	12	(60.0)	
4. Something that is normally effortless was difficult to do					
Not at all	144	(56.3)	112	(43.8)	**0.017**
Sometimes	46	(43.0)	61	(57.0)	
Always	17	(36.2)	30	(63.8)	
Not applicable	8	(42.1)	11	(57.9)	
5. Felt the future is bright					
Not at all	26	(61.9)	16	(38.1)	0.334
Sometimes	38	(47.5)	42	(52.5)	
Always	124	(50.4)	122	(49.6)	
Not applicable	27	(44.3)	34	(55.7)	
6. Felt life so far has been a failure					
Not at all	190	(51.5)	187	(48.5)	0.606
Sometimes/Always	24	(47.1)	27	(52.9)	
7. Felt frightened					
Not at all	171	(54.5)	143	(45.5)	**0.018**
Sometimes	44	(38.3)	71	(61.7)	
8. Could not sleep well					
Not at all	136	(55.3)	110	(44.7)	0.062
Sometimes	60	(45.1)	73	(54.9)	
Always	19	(38.0)	31	(62.0)	
9. Felt happy					
Not at all	20	(45.5)	24	(54.5)	0.827
Sometimes	19	(46.3)	22	(53.7)	
Always	165	(50.9)	159	(49.1)	
Not applicable	11	(55.0)	9	(45.0)	
10. Felt more reserved than usual					
Not at all	148	(50.5)	145	(49.5)	0.981
Sometimes	48	(49.0)	50	(51.0)	
Always	7	(46.7)	8	(53.3)	
Not applicable	12	(52.2)	11	(47.8)	
11. Felt lonely					
Not at all	165	(56.9)	125	(43.1)	**<0.001**
Sometimes/Always	50	(36.0)	89	(64.0)	
12. People around me seem cold to me					
Not at all	191	(51.1)	183	(48.9)	0.226
Sometimes	24	(43.6)	31	(56.4)	
13. Cried or felt like crying					
Not at all	192	(57.3)	143	(42.7)	**<0.001**
Sometimes	23	(24.5)	71	(75.5)	
14. Felt sad					
Not at all	174	(61.7)	128	(45.4)	**<0.001**
Sometimes	41	(32.3)	86	(67.7)	
15. Felt people around me disliked me					
Not at all	195	(51.3)	185	(48.7)	0.254
Sometimes	20	(40.8)	29	(59.2)	
16. Didn’t feel like doing anything					
Not at all	165	(52.1)	152	(47.9)	0.223
Sometimes	50	(44.6)	62	(55.4)	

n = count/frequency.

a = Chi-square test for independence.

Bold value represents significance at 0.05.

Table 4 illustrates the social health among the older adults. Univariate analysis showed that the spouse of male participants (61.0%) would be significantly more concern about them compared to spouse of female participants (39.0%) (*p* < 0.001). Similarly, male participants (59.4%) were significantly more concerned about their spouse if they had problem compared to female participants (40.6%) (*p <* 0.001). Both male and female participants reported visiting friends or relatives equally frequent, at least once a month. In addition, most of them had more than one close friend or relative that they could confide and call for help.

**TABLE 4:** Social health of older persons by gender (National Study of Elderly Person, Brunei, 2019).

Social health	Male (*n =* 215)		Female (*n =* 214)		*p-*value^a^
	N	(%)	N	(%)	
Frequency of meeting relatives or friends					
Almost every day	77	(58.8)	54	(41.2)	0.090
2–3 times a week	54	(50.0)	54	(50.0)	
Once a week	23	(56.1)	18	(43.9)	
Every 2 weeks	27	(42.9)	36	(57.1)	
Once a month	12	(32.4)	25	(67.6)	
Every 2 months or more	22	(44.9)	27	(55.1)	
How many relatives you can talk at ease on private matters					
None	27	(58.7)	19	(41.3)	0.404
One person	17	(47.2)	19	(52.8)	
Two persons	31	(42.5)	42	(57.5)	
Three or four persons	51	(49.0)	53	(51.0)	
Five to eight persons	41	(47.7)	45	(52.3)	
Nine or more	48	(57.1)	36	(42.9)	
How many close relatives you can call for help					
None	27	(54.0)	23	(46.0)	0.853
One person	14	(41.2)	20	(58.8)	
Two persons	35	(46.6)	40	(53.3)	
Three or four persons	49	(52.1)	45	(47.9)	
Five to eight persons	44	(51.2)	42	(48.8)	
Nine or more	46	(51.1)	44	(48.9)	
Who is primarily responsible for following tasks					
1. Care of children					
Self only	10	(16.9)	49	(83.1)	**<0.001**
Primarily self	5	(21.7)	18	(78.3)	
Equally with spouse	162	(61.1)	103	(38.9)	
Primarily spouse	15	(78.9)	4	(21.1)	
Not applicable	23	(36.5)	40	(63.5)	
2. Earn income					
Self only	47	(49.5)	48	(50.5)	**<0.001**
Primarily self	17	(40.5)	25	(59.5)	
Equally with spouse	131	(64.9)	71	(35.1)	
Primarily spouse	12	(20.3)	47	(79.7)	
Not applicable	8	(25.8)	23	(74.2)	
3. Household chores					
Self only	19	(19.8)	77	(80.2)	**<0.001**
Primarily self	5	(10.4)	43	(89.6)	
Equally with spouse	141	(65.6)	74	(34.4)	
Primarily spouse	42	(93.3)	3	(6.7)	
Not applicable	8	(32.0)	17	(68.0)	
4. Care of older relatives					
Self only	11	(31.4)	24	(68.6)	**<0.001**
Primarily self	2	(28.6)	5	(71.4)	
Equally with spouse	74	(67.3)	36	(32.7)	
Primarily spouse	4	(50.0)	4	(50.0)	
Not applicable	124	(46.3)	144	(53.7)	
5. Manage household accounts					
Self only	27	(31.4)	59	(68.6)	**<0.001**
Primarily self	8	(26.7)	22	(73.3)	
Equally with spouse	157	(61.3)	99	(38.7)	
Primarily spouse	14	(50.0)	14	(50.0)	
Not applicable	9	(31.0)	20	(69.0)	
6. Decide on major purchases					
Self only	31	(33.3)	62	(66.7)	**<0.001**
Primarily self	7	(20.6)	27	(79.4)	
Equally with spouse	154	(62.1)	94	(37.9)	
Primarily spouse	17	(60.7)	11	(39.3)	
Not applicable	6	(23.1)	20	(76.9)	

n = count/frequency.

a = Chi-square test for independence.

Bold value represents significance at 0.05.

In terms of caring for children, in situations where the task was equally shared with spouse, male participants (61.1%) reported significantly higher involvement compared to female participants (35.1%). Conversely, in situations where the task of caring for children were done by self or primarily self only, female participants (78.3–83.1%) reported significantly higher involvement compared to male participants (16.9%–21.7%). This was also consistent with further univariate analysis on other tasks including earning income, doing household chores, caring of older relatives, managing household accounts, and deciding on major purchases.

Table 5 presents physical health status of the sample. The results showed that male and female participants were equally suffering from musculoskeletal pain. However, there was significantly higher reports of shoulder pain, back pain, leg pain and knee pain from female participants. It was also observed that female participants (51.7%–70.7%) received significantly higher health services such as home-based nursing and medical assistance devices (for example, wheelchair) compared to male participants (29.3%–48.3%). Participants were equally reporting issues with vision and hearing, kidney, liver and heart problems, high blood pressure, diabetes, and psychiatric disorders.

**TABLE 5:** Physical health of older persons by gender (National Study of Elderly Person, Brunei, 2019).

Physical health	Male (*n =* 215)		Female (*n =* 214)		*p-*value^a^
	N	(%)	n	(%)	
What part of body feel pain and how bad was it?					
1. Headache					
Mild	73	(50.7)	71	(49.3)	0.198
Moderate/Severe	26	(37.1)	44	(62.9)	
No pain	97	(53.9)	83	(46.1)	
Not applicable	19	(54.3)	16	(45.7)	
2. Shoulder					
Mild	38	(41.8)	53	(58.2)	**0.043**
Moderate/Severe	29	(40.8)	42	(59.2)	
No pain	127	(55.0)	104	(45.0)	
Not applicable	20	(57.1)	15	(42.9)	
3. Arm					
Mild	27	(40.3)	40	(59.7)	0.074
Moderate/Severe	24	(42.1)	33	(57.9)	
No pain	140	(53.4)	122	(46.6)	
Not applicable	24	(55.8)	19	(44.2)	
4. Wrist					
Mild	26	(44.1)	33	(55.9)	0.184
Moderate/Severe	21	(52.5)	19	(47.5)	
No pain	145	(51.1)	139	(48.9)	
Not applicable	23	(50.0)	23	(50.0)	
5. Fingers					
Mild	19	(37.3)	32	(62.7)	0.074
Moderate/Severe	18	(42.9)	24	(57.1)	
No pain	154	(53.1)	136	(46.9)	
Not applicable	24	(52.2)	22	(47.8)	
6. Chest					
Mild	25	(42.4)	34	(57.6)	0.502
Moderate/Severe	18	(56.3)	14	(43.8)	
No pain	147	(50.7)	143	(49.3)	
Not applicable	25	(52.1)	23	(47.9)	
7. Stomach ache					
Mild	30	(48.4)	32	(51.6)	0.757
Moderate/Severe	21	(53.8)	18	(46.2)	
No pain	141	(50.2)	140	(49.8)	
Not applicable	23	(48.9)	24	(51.1)	
8. Back					
Mild	43	(39.8)	65	(60.2)	**0.047**
Moderate/Severe	35	(44.9)	43	(55.1)	
No pain	116	(56.3)	90	(43.7)	
Not applicable	21	(58.3)	15	(41.7)	
9. Leg					
Mild	41	(51.3)	39	(48.7)	**0.029**
Moderate/Severe	32	(35.2)	59	(64.8)	
No pain	123	(54.9)	101	(45.1)	
Not applicable	19	(55.9)	15	(44.1)	
10. Knees					
Mild	46	(41.8)	64	(58.2)	**0.008**
Moderate/Severe	42	(40.8)	61	(59.2)	
No pain	110	(58.5)	78	(41.5)	
Not applicable	17	(60.7)	11	(39.3)	
11. Ankle					
Mild	27	(49.1)	28	(50.9)	0.265
Moderate/Severe	20	(36.4)	35	(63.6)	
No pain	147	(52.9)	131	(47.1)	
Not applicable	21	(51.2)	20	(48.8)	
12. Toes					
Mild	22	(42.3)	30	(57.7)	0.673
Moderate/Severe	14	(43.8)	18	(56.3)	
No pain	157	(52.0)	145	(48.0)	
Not applicable	22	(51.2)	21	(48.8)	

n = frequency.

a = Chi-square test for independence.

Bold value represents significance at 0.05.

Table 6 demonstrates the correlates of perceived health and satisfaction with life among the older adults. For male participants, after adjusting for possible confounding factors, it was observed that number of children, estimated income, having chronic illness and diabetes were significantly associated with overall perceived health, explaining 33.1% of the total variance. Increase in number of children was observed to increase the perception of overall health. Conversely, increase in income, chronic illness and diabetes were observed to reduce the perception of overall health. In contrast, for female participants, 38.9% of the variance of overall perceived health could be explained by satisfaction with life and difficulty to do tasks that were normally easy.

**TABLE 6:** Factors associated with perceived health among older person by gender (National Study of Elderly Person, Brunei, 2019).

	Male (*n* = 215)				Female (*n* = 214)			
	Adjusted *b* ((95% CI)		*p* Value	R^2^	Adjusted *b* ((95% CI)		*p* Value	*R* ^*2*^
	Final model				Final model			
1. Perceived health				0.331				0.389
No. of children								
1 to 2 children	—	—	—		—	—	—	
3 to 4 children	0.79	(0.13, 1.46)	0.019		—	—	—	
5 to 6 children	0.69	(0.04, 1.35)	0.038		—	—	—	
>6 children	—	—	—		—	—	—	
Estimated income	−0.09	(−0.17, −0.02)	0.013		—	—	—	
Diagnosed with chronic illness	−0.69	(−1.03, −0.34)	<0.001		—	—	—	
Health problem (diabetes)	−0.98	(−1.58, −0.38)	0.002		—	—	—	
Satisfaction with life	—	—	—		0.32	(0.16, 0.48)	<0.001	
Something that is normally effortless was difficult to do	—	—	—		−0.25	(−0.46, −0.04)	0.018	
2. Satisfaction with life				0.263				0.456
Difficulty to shower by self	−1.42	(−2.48, −0.37)	0.009		—	—	—	
Smoking	0.29	(0.06, 0.52)	0.015		—	—	—	
Concern about health	−0.19	(−0.30, −0.08)	0.001		—	—	—	
Concern towards friends, acquaintances or neighbours	0.25	(0.03, 0.48)	0.028		0.30	(0.02, 0.58)	0.035	
Walking in daily activities	−0.10	(−0.19, −0.01)	0.028		—	—	—	
Leg pain	−0.10	(−0.21, −0.00)	0.046		—	—	—	
Health problem (kidney)	−0.39	(−0.75, −0.03)	0.034		—	—	—	
Education level	—	—	—		−0.08	(−0.17, −0.00)	0.044	
Overall Perceived health	—	—	—		0.43	(0.22, 0.64)	<0.001	
Concern towards spouse	—	—	—		−0.38	(−0.63, −0.14)	0.003	
Concern from spouse	—	—	—		0.52	(0.21, 0.83)	0.002	

Multiple linear regression model *b* = Regression coefficient; *n* = Frequency, CI, Confidence interval.

Bold value represents significance at 0.05.

Multiple regression was also applied for satisfaction with life. For female participants, 45.6% of the variance could be explained by having concern for friends, acquaintances or neighbours, education level, perceived health, and concern towards and from spouse, after adjusting for possible confounding factors. It was observed that higher concern for friends, acquaintances or neighbours, perceived health, and concern from spouse, increases satisfaction with life among female participants. In contrast, 26.3% of the variance for satisfaction with life among male participants could be explained by difficulty to have shower by themselves, smoking, being concern about health, having concern for friends, acquaintances or neighbours, walking as part of daily activity, leg pain and kidney problem. It was observed that smoking and having concern for friends, acquaintances or neighbours increases satisfaction with life among male participants.

## Discussion

To the best of the authors’ knowledge, this is the first paper that reports gender differences of older adults in Brunei. This study discovered several important findings on gender differences among elderly person in Brunei in comparison with different countries worldwide. The rate of aging is rapidly comparable to Japan and Korea [29, 30]. First of all, elderly women generally show lower life satisfaction as they are not only susceptible to chronic illnesses and disabilities, but also receptive to burden associated with traditional female roles [31]. This is consistent, in the present study and previous studies [18, 19], where elderly women reported significantly lower life satisfaction than men. However, there is contradictory results revealed in several countries. In South Korea, elderly men reported lower life satisfaction than women [20]. In China, elderly women reported higher life satisfaction [21]. In Spain, life satisfaction is equally shared between men and women [3].

Life satisfaction is a complex, multi-dimensional phenomenon, and the differences based on gender could possibly be mediated by other predictors such as socioeconomic and sociocultural factors on gender roles, opportunities and disadvantages across life events [31]. In the present study, the highest predictor of life satisfaction among elderly women was positive self-perception of health, similar to a study in Brazil [31], together with other significant social factors including having concern for spouse friends, acquaintances or neighbours. In addition, we also revealed that women were still actively engaged in traditional gender roles such as care of children, household chores, and care of older relatives by themselves. Education level could also play an important part, where those with lower level of education were significantly more satisfied, which is congruent to a study in China [32] but in contrast to a study in Spain where higher education level contributed to higher life satisfaction [3]. For elderly men, there was overlapping predictors with women, however, major differences were variables more incline towards physical activities and abilities. The highest predictor of life dissatisfaction among men was difficulty to perform daily activities, particularly taking shower. In our results, men had significantly lower proportions of musculoskeletal pain or discomfort, and they generally were able to perform daily activities without assistance. This could explain why elderly men reported higher life satisfaction than female. In South Korea, elderly men reported lower level of physical health as well as lower social relationship, which had contributed to lower life satisfaction and increased mental health issues [20, 33].

Healthy aging is tightly intertwined with life satisfaction as shown in our results, similar to prior studies [34–36]. This is particularly evident for older women, as discussed above. The proportion of musculoskeletal discomfort as well as mental health and emotional conditions were significantly higher among older women. The high correlation between physical activity on mental health could explain this relationship. Previous studies have determined effects of exercise on mental health [37, 38], and the current Coronavirus disease 2019 (COVID-19) pandemic might deteriorate the situation further with mandated social isolation and restrictions on public movement [38]. The World Health Organization has recommended at least 150 min per week of moderate-intensity physical activity such as brisk walking and riding a bike, or at least 75 min per week of vigorous-intensity physical activity such as running or fast swimming, for older adults [39]. This include home-based physical activity such as aerobic exercises, bodyweight training, dance and active gaming to counter physical and mental side effects of COVID-19 regulations [40]. Furthermore, social participation has also been a critical indicator of positive health effects on older adults [5]. In Japan, a gender stratified analysis revealed that social participation improved physical and mental health of older women more than men, and overall benefits were recorded for both gender compared to non-participation [5]. Being active in various social activities such as sport groups, hobby clubs, gardening, recreational training, and so forth, have demonstrated positive effects in different older adults [5, 41, 42].

Moreover, in the present study, having more children were perceived as healthier among older men. Even though numerous studies have considered the association between health and number of children, very few have examined gender differences. We could only postulate that, in this population, older men may place importance in intergenerational transfers and support such as practical help and financial aid, while older women on emotional support and care [1]. Another reason might institute from culturally—what constitute healthy or successful aging—defined achievement. This could include pride of continuing the family line, as well as related benefits of self-esteem gained, economic and social security, that might outweigh burden of having fewer or no children such as continuing unwanted involvement in children’s lives, prolonged mental aggravations, and continuing financial demands from children [9].

The results of this study should be interpreted within its limitations. Cross-sectional nature of this study limits prospective causal inferences, and the self-reported data collected is subject to recall and reporting bias. In addition, the small sample size of this study limits generalizability to the study population. Even though we have investigated major domains of life satisfaction and healthy aging for older adults, future studies should include other dimensions that are no less salient such as spiritual experiences and perception of death [43] as well as emerging components such as gerontechnology and digital health, to provide a comprehensive and holistic approach towards successful aging.

In conclusion, this study has made large strides in understanding gender differences in life satisfaction and healthy aging, as well as providing epidemiologic estimates in socioeconomic status, physical health, mental and emotional conditions, and social health status for older men and women in Brunei. However, more efforts from policymakers and related stakeholders are still needed to close gender gaps among the older adults. This can be done by formulating appropriate and localized policies of ensuring living spaces, transportation, and communication services as well as community and other projects that can increase physical activity, social interaction and maintain relationships among older adults.

## Data Availability

The datasets generated and/or analysed during the current study are not publicly available due to institutional data sharing clause but are available from the corresponding author on reasonable request.
